# Our Department of Society Reports

**Published:** 1877-09

**Authors:** 


Our department of Society Reports has not for several months past, been as full as usual, owing mainly to the suspension of the meetings of the Academy of Medicine during the summer months. The exercises will, however, be resumed on the first of October, and the proceedings will again appear in our next issue, and be continued regularly as heretofore.

The discussions of other local Societies in this State will hereafter be reported for publication in this journal.

The reports and discussions in Medical Societies tend greatly to professional and scientific advancement, and we have always felt a lively interest in such associations when conducted for this purpose. Correct and faithful reports must be relied on for the establishment of practical facts, and it would be most outrageous in members to make other than strictly correct statements of facts in their reports. One who would vary from this is not only unworthy of membership in societies, but of professional recognition. Of course opinions, inferences, explanations of phenomena, etc., may differ greatly amongst scientific men, but facts cannot be varied, and should always be stated with great care and correctness.

